# Does Herpes Zoster Increase the Risk of Stroke and Myocardial Infarction? A Comprehensive Review

**DOI:** 10.3390/jcm8040547

**Published:** 2019-04-22

**Authors:** Ping-Hsun Wu, Yun-Shiuan Chuang, Yi-Ting Lin

**Affiliations:** 1 Graduate Institute of Clinical Medicine, College of Medicine, Kaohsiung Medical University, Kaohsiung 807, Taiwan; 970392@kmuh.org.tw; 2 Faculty of Medicine, College of Medicine, Kaohsiung Medical University, Kaohsiung 807, Taiwan; 3 Division of Nephrology, Department of Internal Medicine, Kaohsiung Medical University Hospital, Kaohsiung 807, Taiwan; 4 Department of Family Medicine, Kaohsiung Medical University Hospital, Kaohsiung 807, Taiwan; kinkipag@gmail.com

**Keywords:** herpes zoster, stroke, myocardial infarction, cardiovascular disease

## Abstract

Herpes zoster (HZ) caused by varicella zoster virus (VZV) reactivation is characterized as a vesicular rash of unilateral distribution that can also cause multiple complications; such as post-herpetic neuralgia; ophthalmic zoster; and other neurological issues. VZV can also increase incident hemorrhagic or ischemic complications by causing inflammatory vasculopathy. Thus; emerging epidemiological and clinical data recognizes an association between HZ and subsequent acute strokes or myocardial infarction (MI). This study reviewed published articles to elucidate the association between HZ and cerebrovascular and cardiac events. Individuals exposed to HZ or herpes zoster ophthalmicus had 1.3 to 4-fold increased risks of cerebrovascular events. Higher risks were noted among younger patients (age < 40 years) within one year after an HZ episode. The elevated risk of CV events diminished gradually according to age and length of time after an HZ episode. The putative mechanisms of VZV vasculopathy were also discussed. Several studies showed that the development of herpes zoster and herpes zoster ophthalmicus increased the risks of stroke; transient ischemic attack; and acute cardiac events. The association between VZV infection and cardiovascular events requires further studies to establish the optimal antiviral treatment and zoster vaccination to reduce zoster-associated vascular risk

## 1. Introduction

Herpes zoster (HZ) is an infectious disease induced by the reactivation of the varicella zoster virus (VZV) from its latent state in the sensory ganglia. VZV is a double-stranded DNA alpha herpesvirus that can be transmitted by direct airborne transmission. Primary infection with VZV in childhood is known as chickenpox, and then the latent VZV may develop HZ (shingles) in adults. HZ usually starts with a prodromal phase with severe, sharp radicular pain and paresthesia spreading in the affected dermatomes. It then proceeds via vesicular eruption on an erythematous base in the dermatomes within 48–72 h. During the acute phase, the erythematous maculopapular rash evolves into vesicles, pustules, and finally scabs. The lesions exfoliate for approximately 10 days, and the skin usually returns to its intact state after 2–4 weeks. The lesion is mostly in the thoracic region (>50%), but is also possible in the ophthalmic, cervical, and lumbosacral regions. Herpes zoster ophthalmicus (HZO) involves the ophthalmic branch of the trigeminal nerve and can occur in approximately 10% to 20% of HZ cases [1]. Reactivated VZV can introduce complications of post-herpetic neuralgia, myelitis, meningoencephalitis, and VZV vasculopathy. The association between vascular diseases and VZV reactivation has been as described herein. However, the vascular risk among patient age or post-herpes zoster duration remains poorly elucidated. The aim of this review is to determine the correlation between HZ or HZO and the occurrence of cerebrovascular and cardiac events on age stratification and various post-herpes zoster time periods.

## 2. Studies Investigating the Association Between HZ and CVD or Cerebrovascular Disease

Comprehensive searches of electronic databases (PubMed, Scopus, Google Scholar, and the Cochrane Library) were performed (date from database inception to February 28, 2019) to identify relevant studies written in English. Text headings and medical subject heading (MeSH) terms used in the searches included HZ, shingles, HZO, stroke, TIA, cerebrovascular disease, and MI. Fifteen studies examined the associations between unspecified HZ types and the following: Non-specified stroke [2,3,4,5,6,7,8,9], ischemic stroke [7,10,11], hemorrhagic stroke [7], transient ischemic attack (TIA), a composite of stroke and TIA [12,13], myocardial infarction (MI) [2,5,9,10,11], acute coronary syndromes (MI and unstable angina) [14], and incident coronary artery disease, including angina and MI (Table 1, Table 2) [15]. There were also eight studies regarding HZO and cardiovascular disease (CVD), five studies of non-specified stroke [2,3,6,7,16], and studies with ischemic stroke [10], hemorrhagic stroke [7], and MI [10] as endpoints (Table 3). The follow-up time periods ranged from 1 week to 24 years. Six meta-analysis studies were also enrolled to investigate the correlation between HZ and cerebrovascular risk [17,18,19,20,21,22].

## 3. Herpes Zoster and Risk of Stroke or Myocardial Infarction

Recent epidemiological studies (*n* = 15) from Asia (Taiwan [3,14,15,16] and Korea [9,11,12]), Iran [8], Europe (the United Kingdom [2,6], Germany [7], Demark [13], and Sweden [4]), and the United States [5,10] showed an increased incidence of stroke or MI in patients with a recent history of zoster. The characteristics of the 15 studies are displayed in Table 1, Table 2 and Table 3. Different databases were used in these studies, including the Taiwanese National Health Insurance Research Database (NHIRD) [3,14,15,16], the United Kingdom’s Clinical Practice Research Datalink (CPRD) and the Health Improvement Network (THIN) general practice databases [2,6], the Danish registry [13], the Swedish registry [4], the German Pharmacoepidemiological Research Database (GePaRD) [7], the US Medicare Database [10] and Olmsted County residents [5], and the Korean Health Insurance Database [9,11,12]. The study designs included a case-control study [8], retrospective cohort studies [2,3,4,5,11,12,13,14,15,16], a propensity score-matching approach [9], and self-controlled case series analyses [6,7,10] with adjustment for age, sex, and CVD risk factors (for example, hypertension diabetes, congestive heart failure, dyslipidemia, ischemic heart disease, atrial fibrillation, intermittent arterial claudication, carotid stenosis, and valvular heart disease). Among these studies, Sundström et al. [4] used only age and sex for adjustment, and Breuer et al. [2] and Langan et al. [6] further controlled for smoking and obesity. Schink et al. [7] and Minassian et al. [10] used a self-controlled case series design that made within-person comparisons to control for confounders (Table 1, Table 2 and Table 3).

The earliest studies used the Taiwan NHIRD to examine the association between HZ and cerebrovascular events. Kang et al. [3] reported a 30% increase in CVD risk (hazard ratio (HR), 1.31; 95% confidence interval (CI), 1.06–1.60) after HZ episode and a four folds increased risk (HR, 4.28; 95% CI, 2.01–9.03) after an HZO episode within one year. Lin et al. [16] found that HZ was a significant risk factor for ischemic stroke (HR, 4.52; 95% CI, 2.45–8.33), but not for hemorrhagic stroke. Sreenivasan et al. [13] also reported an increased risk of stroke after HZ episodes in a Denmark population-based cohort study between 1995 and 2008. A total of 4876 subjects had a stroke during the follow-up period among 117,926 HZ patients. An increased risk of stroke after treatment for herpes zoster was observed for the following one year (incident rate ratio (IRR), 2.27; 95% CI, 1.83–2.82). Using the 2002–2010 THIN Database, Breuer et al. [2] evaluated the risk of stroke, TIA, and MI in 106,601 HZ patients with one episode of herpes zoster with 213,202 controls matched for age, sex, and general practice. Increased risks of MI (HR, 1.10; 95% CI, 1.05–1.16) and TIA (HR, 1.15; 95% CI, 1.09–1.21) were found. The risk was highest in patients under 40 years of age as risk of stroke, TIA, and MI (HR, 1.74, 2.42, and 1.49). The risk disappeared in the analysis of all age populations (HR, 1.02; 95% CI, 0.98–1.07). Sundström et al. [4] reported a 1.34-fold (IRR, 1.34; 95% CI, 1.12–1.62) increased risk of stroke within 1 year after HZ episodes in all age groups in a Swedish register-based cohort controlling for age and sex. Minassian et al. [10] used the Medicare Database to study subjects more than 65 years old with an HZ diagnosis between 2006 and 2011. Among them, 42,954 had incident ischemic stroke and 24,237 had incident MI. In a self-controlled case series, a 2.4-fold increased rate of ischemic stroke (IRR, 2.37; 95% CI, 2.17–2.59) and a 1.7-fold increased rate of MI (IRR, 1.68; 95% CI, 1.47–1.92) were reported during the first week after an HZ episode. However, the risk of both incidents gradually decreased during the following 6 months. Because of limited power, this study showed no evidence of MI or stroke prevention by vaccination against zoster [10]. Previous three studies using the Korean Health Insurance Database found increased risks of stroke, TIA, and MI in HZ patients. Kwon et al. [12] reported higher risk of stroke/TIA (IRR, 1.90; 95% CI, 1.85–1.95) and Kim et al. [9] further found that HZ was associated with a 35% increased risk of stroke (HR 1.35; 95% CI, 1.18–1.54) and a 59% increased risk of MI (HR 1.59; 95% CI, 1.27–2.01) in a propensity score-matched analysis. In addition, Seo et al. [11] showed that severe HZ requiring hospitalization increased the risk of MI (HR, 1.83; 95% CI, 1.35–2.48) and ischemic stroke (HR, 1.52; 95% CI, 1.21–1.92). In the USA, Yawn et al. [5] compared 4862 individuals older than 50 years versus 19,433 control matched individuals and found a recent episode of herpes zoster was associated with a higher risk of stroke or MI. They found a greater risk of stroke at 3 months’ post-HZ (odds ratio (OR), 1.53; 95% CI, 1.10–2.33), but the association between HZ and MI at 3 months was not robust in a sensitivity analysis. According to the meta-analysis results reported by Yang et al. [19], the combined relative risk (RR) was 1.36 (95% CI, 1.10–1.67), which demonstrated that HZ patients had a 36% greater risk of developing stroke (Table 4). The analysis also identified that HZ was associated with ischemic stroke (RR, 1.99; 95% CI, 1.04–3.81), but not hemorrhagic stroke (RR, 1.86; 95% CI, 0.76–4.54). Recently, Patterson et al. [23,24] estimated the risk of TIA, stroke, MI, and composite vascular events using the Market Scan Commercial and Medicare 2007 to 2014 dataset linked with Electronic Medical Records (EMR). A 1.56-fold increase risk of TIA (95% CI, 1.13–2.15) and a statistically insignificant 1.40-fold increase of stroke (95% CI, 0.93–2.11) in 23,339 HZ subjects compared to 46,378 propensity-matched (sociodemographic and clinical factors) controls [24]. The adjusted IRR disclosed a 1.3-fold increase in composite vascular events (95% CI, 1.03–1.65) in the HZ group [23]. In summary, the incidence rate of post herpes zoster associated cardiovascular events are 9.56 to 17.98 per 1000 person-years in stroke/TIA [11,12,15] and 3.68 to 5.47 per 1000 person-years in myocardial infarction [11,14]. There was a significant association between HZ and stroke or MI in these epidemiology studies. Regarding the stroke subtype, an increased risk was observed in ischemic stroke, but not in hemorrhagic stroke after HZ infection [18].

## 4. Herpes Zoster Ophthalmicus and Risk of Stroke

HZO is HZ reactivation in the ophthalmic branch of the trigeminal nerve, the nerve that provides sensation to the eyes and forehead. Kang et al. [3] and HR, 4.52; 95% CI, 2.45–8.33 in Lin et al. [16] Antiviral treatment for zoster had no effect on the incidence of subsequent cerebral vascular disease [16]. Langan et al. [6] reported a 3-fold increased risk of stroke from 5–12 weeks following HZO (IRR, 3.38; 95% CI, 2.18–5.24). However, Breuer et al. [2] found a non-significant risk of stroke post-HZO (HR, 1.03; 95% CI, 0.77–1.39) using UK databases (CPRD and THIN) to investigate the association between HZO and stroke risk. Using the GePaRD, Schink et al. [7] performed a self-controlled case series study to demonstrate a 1.5 times higher risk of stroke 3 months after an HZO episode (IRR, 1.59; 95% CI, 1.10–2.32). In this study, the risk of ischemic stroke increased (IRR, 1.57; 95% CI, 1.05–2.35) among HZO patients, but not hemorrhagic stroke (IRR, 1.82; 95% CI, 0.62–5.37) [7] A similar finding was presented by Minassian et al. [10] of a higher ischemic stroke risk the first week (IRR, 2.73; 95% CI, 2.22–3.35) and three months (IRR, 1.29; 95% CI, 1.15–1.44) after an HZO episode. However, Yang et al. [19] included three studies [2,3,16] in a meta-analysis to demonstrate increased stroke risk related to HZ, and the pooled RR was 2.62 (95% CI, 0.85–8.06), but the heterogeneity was high (Table 1).

## 5. Post-Herpes Zoster Length of Follow-Up and Age Stratification with Associated Risk of CVD

**Figure 1:**
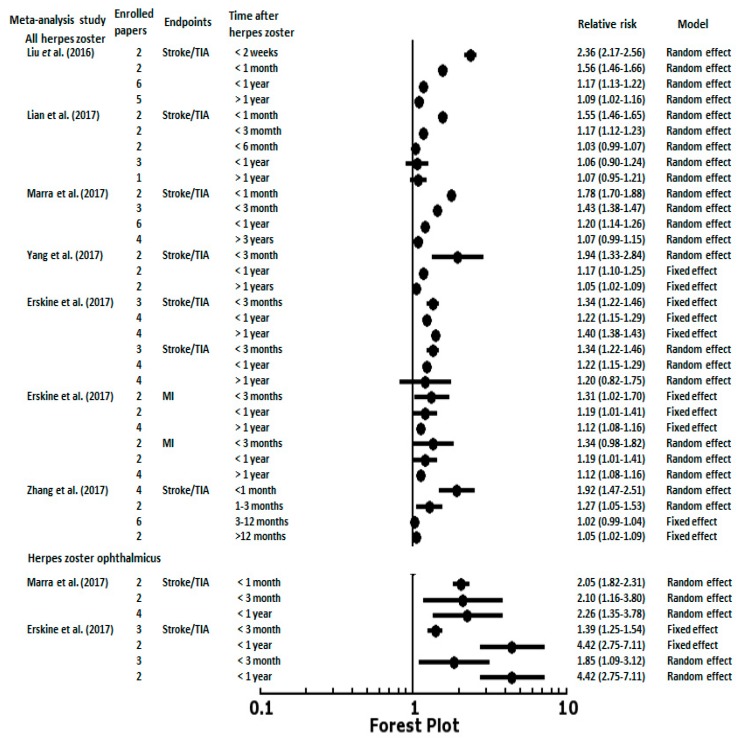
The results of meta-analyses examining the relative risk (RR) of stroke within different lengths of follow-up after herpes zoster episodes. TIA, transient ischemic attack; MI, myocardial infarction.

The associations between post-HZ length of follow-up and CVD or cerebrovascular events were noted in several epidemiology studies designed as self-controlled case series and meta-analysis reports. The temporal pattern of the risk of stroke was similar in Langan et al. [6], Minassian et al. [10], and Schink et al. [7]. All three studies were based on a self-controlled case series study design. They found that the risk of stroke increased in the first week after an HZ episode then decreased after 6 to 12 months. The peak of the risk of stroke observed by Minassian et al. [10] was within the first weeks (IRR, 2.37; 95% CI, 2.17–2.59) after HZ onset, followed by a gradual decline in the risk (IRR, 1.55; 95% CI, 1.46–1.66 at 2–4 weeks; IRR, 1.17; 95% CI, 1.11–1.22 at 5–12 weeks). Sreenivasan et al. [13] enrolled 4.6 million people in Denmark and revealed a 127% (IRR, 2.27; 95% CI, 1.83–2.82) increase in the risk of stroke within two weeks after an HZ episode, and a 17% (IRR, 1.17; 95% CI, 1.09–1.24) increased risk from 2 weeks to 1 year after an HZ episode. Langan et al. [6] also found that the risk varied according to the time period after HZ with an incidence of 1.63 at 1–4 weeks, 1.42 at 5–12 weeks, and 1.23 at 13–26 weeks after an HZ episode. Regarding the antiviral treatment effect, 55% of HZ patients with antiviral treatment had a reduced stroke risk. However, a direct comparison cannot be made because of the diversity of HZ onset definition in the different studies. For example, Sreenivasan et al. [13] defined HZ onset as the start of antiviral therapy. Minassian et al. [10] used the start of antiviral therapy within 7 days before or after HZ diagnosis. Schink et al. [7] defined HZ onset as admission to hospital or the start of antiviral therapy. Kang et al. [3], Lin et al. [16], and Langan et al. [6] assessed the HZ diagnosis by International Classification of Diseases (ICD) codes. Therefore, the meta-analysis data demonstrated more consistent patterns and results of CVD and cerebrovascular events stratified by length of follow-up after HZ episodes. Liu et al. [17] used data from eight studies to show a gradient of stroke risk decreasing from 2.36 (95% CI, 2.17–2.56) in the first 2 weeks after an HZ episode to 1.56 (95% CI, 1.46–1.66) at 1 month, 1.17 (95% CI, 1.13–1.22) at 1 year, and 1.09 (95% CI, 1.02–1.16) after 1 year (Figure 1). Lian et al. [18] meta-analyzed eight studies and found the short-term risks increased for ischemic stroke after an HZ episode, whereas it was not found in the long-term follow-up. The pooled relative ratios (RRs) for ischemic stroke after an HZ episode were 1.55 (95% CI, 1.46–1.65) within one month, 1.17 (95% CI, 1.12–1.23) within three months, and 1.03 (95% CI, 0.99–1.07) within six months (Figure 1). Similar results were found by Marra et al. [20], indicating that the RR for stroke was 1.78 (95% CI, 1.70–1.88) in the first month, 1.43 (95% CI, 1.38–1.47) after three months, 1.20 (95% CI, 1.14–1.26) after 1 year, and no relationship was found after three years (RR, 1.07; 95% CI, 0.99–1.15). Three meta-analysis studies assessed the association between HZ and stroke/TIA or MI at different time periods after an HZ episode (Figure 1). Similar time period stratification (<3 months, <1 year, and >1 year) and random effect meta-analysis results were noted. The pooled RRs for the HZ-associated risk of stroke/TIA presented decreasing gradients of risk from short-term periods of time (<3 months and <1 year) to long-term periods of time (>1 year) [19,21,22]. The RRs for stroke/TIA post-HZ within three months were 1.94 (95% CI, 1.33–2.84) in Yang et al. [19], 1.34 (95% CI, 1.22–1.46) in Erskine et al. [21], and 1.27 (95% CI, 1.05–1.53) in Zhang et al. [22]. The RRs within one year were 1.17 (95% CI, 1.10–1.25) in Yang et al. [19], 1.22 (95% CI, 1.15–1.29) in Erskine et al. [21], and 1.02 (95% CI, 0.99 to 1.04 observed from 3 to 12 months) in Zhang et al. [22] To manage the statistical heterogeneity, Erskine et al. [21] reported the risks of stroke at one year after an HZ episode were significant in the fixed effects model (RR, 1.40; 95% CI, 1.38–1.43), but not in the random effects model (RR, 1.20; 95% CI, 0.82–1.75). Regarding MI, patients with HZ had higher risks of cardiac events at three months using the fixed effects model (RR, 1.31; 95% CI, 1.02–1.70), but not the random effects model (RR, 1.34; 95% CI, 0.98–1.82). The risks of cardiac events at one year and over one year after an HZ episode were higher using the fixed and random effects models (RR, 1.19; 95% CI, 1.01–1.41; RR, 1.12; 95% CI, 1.08–1.16, respectively) (Figure 1). The increased risk of stroke post-HZO at different periods of time was observed in two meta-analysis studies reported by Marra et al. [20] and Erskine et al. [21]. Due to the limited numbers of HZO cases and observation times, the RRs were 1.3–4.4-fold higher risks of stroke/TIA after HZO in short-term periods of time (<1 month, <3 months, and <1 year) (Figure 1).

Since differences in the choice of controls, periods for between-group comparisons, and controlling for confounders greatly affect outcomes, the results of these studies are hardly comparable. Considering the more reliable method by self-controlled case series analyses, three studies (Langan et al., Minassian et al., and Schink et al.) [6,7,10] confirmed the results of a higher incidence ratio of post herpes zoster associated cardiovascular events in different risk periods after HZ diagnosis. The age-adjusted incidence ratios of ischemic stroke were 1.30–2.37 within 4 weeks, 1.17–1.42 during 5–12 weeks, and no effect was found after 12 weeks [6,7,10]. Similarly, the risk of MI after an HZ episode was increased within 12 weeks and stabilized after 12 weeks [10] (Table 5). The risks of cerebrovascular or cardiovascular complications associated with HZ appear transient in nature, tending to decrease with time, and returning to general population risks by 12 weeks after HZ based on self-controlled case series analyses. These studies have the major benefit of implicitly controlling for fixed between-person confounding effects. However, most prospective cohort studies show a gradient of stroke risk up to 1 year [4,5,13]. More research on the transient increased vascular risks post HZ is needed to guide strategies and policies for risk prevention.

**Figure 2:**
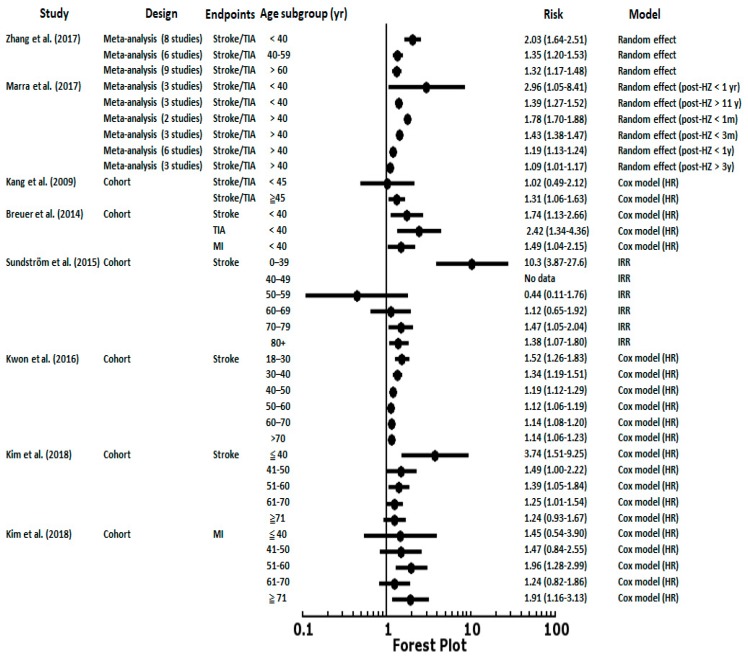
The results of meta-analyses and enrolled cohorts examining the risk of stroke or myocardial infarction after herpes zoster episodes in different age ranges. TIA, transient ischemic attack; MI, myocardial infarction; HR, hazard ratio; IRR, incident rate ratio.

Figure 2 shows the risks of stroke/TIA and MI after an HZ episode in different age groups. As the Zhang et al. [22] and Marra et al. [20] meta-analyses reported, there was a significant increased risk of stroke after an HZ episode in younger subjects. The risk of stroke showed a decreased gradient from younger to elderly patients as in Kwon et al. [12] and Kim et al. [9]. In Breuer et al. [2] and Sundström et al. [4], the risks of CVD or cerebrovascular disease were especially high in patients <40 years, a group with fewer atherosclerosis risk factors. These findings were also compatible with recent studies by Patterson et al. [23,24] that found 5 times (95% CI, 1.37–19.10) higher risks of TIA and 3 times (95% CI, 1.15–7.57) higher risks of composite vascular events (MI, ITA, and stroke) in adults aged <50 years. These studies reported that VZV is a risk factor for stroke, especially in individuals under 50 years of age who develop HZ.

**Figure 3:**
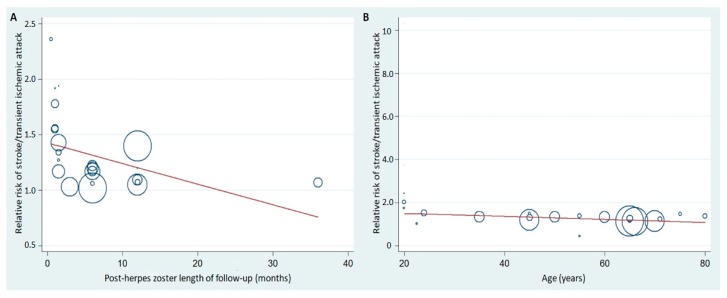
Meta-regression of the post-HZ length of follow-up (**A**) or age of the subjects (**B**) and relative risk of stroke/TIA in post-HZ patients. HZ, herpes zoster.

Figure 3 demonstrates the meta-regression of the post-HZ length of follow up as well as age on stroke/TIA risk. Each circle represents a study subgroup and the size of the circle reflects the influence of that study subgroup on the model (inversely proportionate to the SE of that study). Post-HZ length of follow-up and age influenced the relative risk of stroke/TIA in post-HZ individuals.

## 6. Varicella Zoster Virus Vasculopathy and Other Potential Mechanisms of Herpes Zoster, Such as CVD Risks

Several studies declared that the vasculopathy caused by VZV was associated with the increased risk of future CV outcomes. A clinical case series found that patients with symptoms of HZ and stroke or TIA presented positive for VZV DNA and anti-VZV antibodies from cerebrospinal fluid samples [25,26]. In a pathology survey, arterial biopsies containing intranuclear Cowdry A inclusions in HZ patients concurrent with stroke were consistent with VZV [27]. Moreover, the brain MRI was abnormal in 97% of VZV vasculopathy cases, frequently revealing lesions at the gray-white matter junctions, deep-seated, and cortical infarctions, and the arteries were involved in 70% of patients as determined by angiography [28].

**Figure 4:**
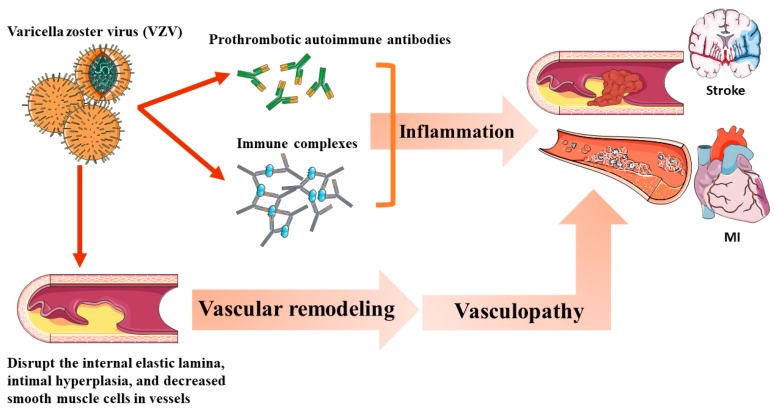
The potential mechanism of varicella zoster virus (VZV)-associated vascular events.

The potential mechanism of VZV-associated vascular events is shown in Figure 4. VZV vasculopathy caused by the productive virus infection of the cerebral arteries can cause pathological vascular remodeling [25], resulting in clinical ischemic or hemorrhagic stroke. HZ induces vasculopathy via the following mechanisms: (1) Induction of the production of prothrombotic autoimmune antibodies, such as IgM and IgG anticardiolipin antibodies [29]. (2) Autoimmune phenomenon caused by circulating immune complexes [30]. (3) Disruption of the internal elastic lamina, intimal hyperplasia, and decreased smooth muscle cells in the tunica medial layer [25].

Virus-induced inflammation accounts for remodeling of the vessel wall that causes an overly thickened intima and inflammation in the vasa vasorum vessels [31,32]. VZV and neutrophils were identified in the arterial adventitia in early vasculopathy. Viral inclusions, DNA, and antigens can be found in the cerebral arteries of VZV vasculopathy in stroke or TIA patients [27,33]. VZV vasculopathy should always be suspected in HZ patients with concurrent TIA, stroke, and/or chronic headache [34]. Most VZV vasculopathies develop within 6 weeks after an HZ episode [35].

The virus sometimes travels from the same trigeminal ganglion along afferent fibers around the carotid artery and its branches [36]. VZV adjacent to small and large intracranial cerebral arteries or extracranial arteries can produce inflammation and pathological vascular remodeling with intima proliferation and media damage, leading to subsequent thrombosis and rupture, culminating in ischemia or hemorrhage [27,36,37,38]. Therefore, VZV can cause cerebrovascular infarctions, aneurysms, and hemorrhages by weakening the cerebral artery walls [27,39]. Moreover, VZV in the cranial nerves has a more direct effect on the cerebral arteries [40]. Stroke patients after an HZ episode showed changes in both brain imaging and angiograms [26,39], which showed vasculitis and lymphocytic infiltration pathology presentation in the infarcted brain [41]. Regarding the pathogenesis of stroke or TIA after an HZ episode located outside of the head and neck, there are several possible biological causes, including (1) an increase in the sympathetic tone, blood pressure, and adverse emotional reactions [42]; (2) altered immunological status caused by VZV reactivation and subsequent vulnerability to cerebrovascular events [42]; and (3) systemic inflammation, autoimmune responses, or hemodynamic changes, leading to cardiovascular events [42].

Vascular events occurring within days of an HZ episode could be due to the associated inflammatory response [43]. For example, inflammatory cytokines, such as interleukin-6 (IL-6), are significantly increased in VZV infections that are related to arterial thrombosis [44,45]. There are different etiologies in ischemic stroke and hemorrhagic stroke, and inflammation plays an important role in the etiology of ischemic stroke. Inflammatory cells could contribute to vascular remodeling by secreting soluble factors and potentially disrupting pre-existing atherosclerotic plaques [46]. In recent studies, the asymptomatic reactivation of VZV from the cranial nerves could also lead to infection of the cranial arteries, stroke, and TIA, including in the absence of HZ rash or rash in the noncranial dermatomes [40]. This hypothesis is supported by findings from a VZV-infected animal model of the simian varicella virus in macaques [47]. In this study, the asymptomatic reactivation of the simian varicella virus with the potential to cause cranial arteritis from the trigeminal ganglia was detected in macaques [48]. In a human study, it was shown diabetes patients without a history of HZ can also have VZV antigen in the arterial adventitial tissue within skip lesions of cerebral arteries [49]. Conclusively, individuals who are predisposed to HZ have a higher risk of cerebrovascular disease.

## 7. Limitations of Current Observational Studies

The limitations of the observational studies included a lack of randomization and a risk of bias. The review studies were controlled for important confounders, such as demographics and CVD risk factors. Some studies were flawed by a small number of enrolled subjectof HZ (Hosamirudsari et al.) [8], few HZO identified patients (Langan et al.) [6], small events numbers (Kang et al., Lin et al., and Sundström et al.) [3,4,16], and misclassification of HZ and herpes simplex (Sreenivasan et al.) [13]. Furthermore, several studies implemented a self-controlled case series design to provide stronger confounding control [6,7,10]. However, the possibility of residual confounding, such as stressful life events or mental health, could not be ruled out. Furthermore, few of the studies evaluated stroke risk according to use of antiviral treatment, which may solidify the evidence of risk association and provide clinical recommendations for adults with high CVD risk after HZ. Correspondingly, only one study evaluated the effect of vaccination on stroke risk after an HZ episode with low power [10]. Additional studies of CVD outcomes among patients vaccinated against HZ could provide a better understanding of HZ as a possible risk factor for CVD. Future cohort studies or clinical trials of patients who received the HZ vaccine could better clarify the association between HZ and CVD or cerebrovascular outcomes. The risk of CVD may depend on whether the HZ outbreak occurs in dermatomes that share innervation with the coronary and cerebral arteries. Although the meta-analysis results demonstrated the association between HZ or HZO and stroke, heterogeneity was observed in the study designs, geographic areas, characteristics of populations, and variant confounding factors’ adjustment. Furthermore, positive result bias may reveal an association between HZ and selected CVD events.

## 8. Conclusions

To summarize the association of HZ and CVD, 15 published epidemiological studies and six meta-analysis reports were reviewed. Individuals exposed to HZ or HZO had 1.3 to 4-fold increased risks of cerebrovascular events. Higher risks were noted among younger patients (age < 40 years) within one year after an HZ episode. The elevated risk of CV events diminished gradually according to age and length of time after an HZ episode. VZV infection of the cerebral arteries with subsequent inflammation leads to vascular remodeling and thickened intima that contributes to vascular occlusion in patients with VZV vasculopathy. Epidemiological studies and the pathology of VZV vasculopathy both indicate that HZ is an important risk factor for stroke within one year after an HZ episode. However, limited reports indicated that antiviral treatment reduces stroke risk. It was also difficult to confirm the relationship between the zoster vaccine and cerebrovascular events because of the small sample size. Emerging studies are needed to determine the effect of optimal antiviral treatment and zoster vaccination on reducing the burden of cerebrovascular and CVD, especially for patients with pre-existing high CVD risk.

## Figures and Tables

**Table 1:** Studies that investigated herpes zoster and the risk of stroke or transient ischemic attack.

Author	Year	Country	Study Design	Study Period	Age (y)	Follow-up (y)	Sample Size	Controlled Factors
Kang et al.	2009	Taiwan	Retrospective matched cohort (age and sex matched)	1997–2001	≧18 (mean:46.7)	1	31,040	Age, sex, hypertension, diabetes, coronary heart disease, hyperlipidemia, renal disease, atrial fibrillation, heart failure, heart valve/myocardium disease, carotid/peripheral vascular disease, monthly income, urbanization level, and geographical region
Sreenivasan et al.	2013	Denmark	Retrospective cohort	1995–2008	≧18	13	4,620,980	Age, sex, calendar period, acute MI, atrial fibrillation, education, cancer, medications (antihypertensives, drugs used to treat dyslipidemia and atrial fibrillation, and immunosuppressive drugs)
Breuer et al.	2014	UK	Retrospective matched cohort (age and sex matched)	2002–2010	≧18 (mean:57.8)	23.7 (median:6.3)	319,803	Age, sex, obesity, smoking, high cholesterol recording, hypertension, diabetes, ischemic heart disease, atrial fibrillation, intermittent arterial claudication, carotid stenosis, and valvular heart disease
Langan et al.	2014	UK	Self-controlled case series	1987–2012	≧18 (median:77)	25 (median:12.5)	6584	Confounders are implicitly controlled for due to the study design
Sundström et al.	2015	Sweden	Retrospective cohort	2008–2010	≧0	1	1.5 million	Age and sex
Minassian et al.	2015	USA	Self-controlled case series	2006–2011	≧65 (median:81.1)	5 (median)	42,954	Confounders are implicitly controlled for due to the study design
Kwon et al.	2016	Korea	Retrospective matched cohort (age matched)	2003–2013	≧18 (mean:41.4)	11	766,179	Age, male gender, hypertension, hyperlipidemia, ischemic heart disease, diabetes, heart failure, peripheral vascular disease, arterial fibrillation or atrial flutter, renal disease, and valvular heart disease
Yawn et al.	2016	USA	Retrospective matched cohort (age and sex matched)	1986–2011	≧50 (mean:68.2)	28.6 (mean:7.1)	24,295	Age, sex, hypertension, dyslipidemia, coronary artery disease (including MI), arrhythmias, congestive heart failure, diabetes, depression, chronic obstructive pulmonary disorder, vasculopathies, stroke, and anxiety
Schink et al.	2016	Germany	Self-controlled case series	2004–2011	≧0 (mean:71.3)	1	124,462	Confounders are implicitly controlled for due to the study design
Hosamirudsari et al.	2018	Iran	Case-control	2015–2017	66.99 (mean)	0.5	210	Age, sex, and hypertension
Kim et al.	2018	Korea	Retrospective propensity-matched cohort	2003–2013	Not reported	11	519,880	Age, sex, body mass index, obesity, smoking, drinking, exercise, economic class, hypertension, diabetes, dyslipidemia, angina pectoris, transient ischemic attack, heart failure, atrial fibrillation/flutter, valvular heart disease, chronic renal disease, carotid stenosis, peripheral vascular disease, chronic liver disease, rheumatoid disease, inflammatory bowel disease, malignancy, transplantation, HIV, and depression
Seo et al.	2018	Korea	Retrospective matched cohort (age and sex matched)	2006–2013	≧40 (mean:63.1 in hospitalized cases and 58 in non-hospitalized cases	8	104,191	Age, sex, income, diabetes mellitus, hypertension, and dyslipidemia

**Table 2:** Studies that investigated herpes zoster and the risk of myocardial infarction.

Author	Year	Country	Study Design	Study Period	Age (y)	Follow-up (y)	Sample Size	Controlled Factors
Wang et al.	2014	Taiwan	Retrospective matched cohort	1999–2010	≧0	12	289,790	Age, sex, urbanization, monthly income, occupation, frequency of medical visits, hypertension, diabetes mellitus, hyperlipidemia, cerebral vascular disease, chronic obstructive pulmonary disease, renal function, cancer, and medication
Breuer et al.	2014	UK	Retrospective matched cohort	2002–2010	≧18 (mean:57.8)	24 (median:6.3)	319,803	Age, sex, obesity, smoking, high cholesterol recording, hypertension, diabetes, ischemic heart disease, atrial fibrillation, intermittent arterial claudication, carotid stenosis, and valvular heart disease
Wu et al.	2015	Taiwan	Retrospective matched cohort	1998–2010	≧20 (mean:46.4)	10	97,415	Age, sex, diabetes, hypertension, and hyperlipidemia
Minassian et al.	2015	USA	Self-controlled case series	2006–2011	≧65 (median:80.3)	5 (median)	24,237	Confounders are implicitly controlled for due to the study design
Yawn et al.	2016	USA	Retrospective matched cohort (age and sex matched)	1986–2011	≧50 (mean:68.2)	28 (mean:7)	24,295	Age, sex, hypertension, dyslipidemia, coronary artery disease (including MI), arrhythmias, congestive heart failure, diabetes, depression, chronic obstructive pulmonary disorder, vasculopathies, stroke, and anxiety
Kim et al.	2018	Korea	Retrospective propensity-matched cohort	2003–2013	Not reported	11	519,880	Age, sex, body mass index, obesity, smoking, drinking, exercise, economic class, hypertension, diabetes, dyslipidemia, angina pectoris, transient ischemic attack, heart failure, atrial fibrillation/flutter, valvular heart disease, chronic renal disease, carotid stenosis, peripheral vascular disease, chronic liver disease, rheumatoid disease, inflammatory bowel disease, malignancy, transplantation, HIV, and depression
Seo et al.	2018	Korea	Retrospective case–control study (age and sex matched)	2006–2013	≧40 (mean:63.1 in hospitalized cases and 58 in non-hospitalized cases	8	104,191	Age, sex, income, diabetes mellitus, hypertension, and dyslipidemia

**Table 3:** Studies that investigated herpes zoster ophthalmicus and the risk of cardiovascular or cerebrovascular disease.

Author	Year	Country	Study Design	Study Period	Age (y)	Follow-up (y)	Sample Size	Endpoints	Confounders (Adjusted for)
Kang et al.	2009	Taiwan	Retrospective matched cohort (age and sex matched)	1997–2001	≧18 (mean:46.7)	1	31,040	Stroke	Age, sex, hypertension, diabetes, coronary heart disease, hyperlipidemia, renal disease, atrial fibrillation, heart failure, heart valve/myocardium disease, carotid/peripheral vascular disease, monthly income, urbanization level, and geographical region
Lin et al.	2010	Taiwan	Retrospective matched cohort (age and sex matched)	2003–2005	≧18 (mean:56.9)	1	2632	Stroke/TIA	Age, sex, hypertension, diabetes, hyperlipidemia, coronary heart disease, chronic rheumatic heart disease, other forms of heart disease, and medication habits
Minassian et al.	2015	USA	Self-controlled case series	2006–2011	80 (median)	6 (median:5)	6971	Stroke	Confounders are implicitly controlled for due to the study design
							3946	MI	Confounders are implicitly controlled for due to the study design
Breuer et al.	2014	UK	Cohort	2002–2010	≧18 (mean:57.8)	24 (median:6.3)	2324	Stroke	Age, sex, obesity, smoking, high cholesterol recording, hypertension, diabetes, ischemic heart disease, atrial fibrillation, intermittent arterial claudication, carotid stenosis, and valvular heart disease
Langan et al.	2014	UK	Self-controlled case series	1987–2012	≧0 (median:77)	25 (median:12.5)	6584	Stroke	Confounders are implicitly controlled for due to the study design
Schink et al.	2016	Germany	Self-controlled case series	2004–2011	≧ 0	1	124,462	Stroke	Confounders are implicitly controlled for due to the study design

**Table 4:** Summary of meta-analysis results of herpes zoster and herpes zoster ophthalmicus with stroke or myocardial infarction.

Study	Year	Herpes Zoster Type	Enrolled Papers	Endpoints	Relative Risk (95% CI)	Model
Yang et at.	2017	All herpes zoster	6	Stroke/TIA	1.36 (1.10–1.67)	Random effect
		Herpes zoster ophthalmicus	3	Stroke/TIA	2.62 (0.85–8.06)	Random effect
Zhang et al.	2017	All herpes zoster	5	Stroke/TIA	1.30 (1.17–1.46)	Random effect
		All herpes zoster	6	MI	1.18 (1.07–1.30)	Random effect
		Herpes zoster ophthalmicus	8	Stroke/TIA	1.91 (1.32–2.76)	Random effect

**Table 5:** Age-adjusted incidence ratios for stroke/TIA or myocardial infarction in length of follow-up time after herpes zoster in three different self-controlled case series studies.

^#^ Time	Langan et al.	Schink et al.	Minassian et al.	Minassian et al.
	Stroke/TIA	Stroke/TIA	Stroke/TIA	Myocardial Infarction
1 week			2.37 (2.17–2.59) *	1.68 (1.47–1.92) *
2 weeks		1.30 (1.00–1.68) *		
3–4 weeks	1.63 (1.32–2.02) *	1.52 (1.20–1.91 ) *	1.55 (1.46–1.66) *	1.25 (1.14–1.37) *
5–12 weeks	1.42 (1.21–1.68) *	1.24 (1.08–1.42) *	1.17 (1.11–1.22) *	1.07 (1.00–1.14) *
13–26 weeks	1.23 (1.07–1.42) *	1.09 (0.97–1.24)	1.03 (0.99–1.07)	1.02 (0.96–1.07)
27–52 weeks	0.99 (0.88–1.12)	0.96 (0.87–1.06)	1.00 (0.96–1.03)	1.02 (0.98–1.07)

* *p* < 0.05, ^#^ Length of follow-up time after herpes zoster episode.
